# Comparison of the source and prognostic utility of cfDNA in trauma and sepsis

**DOI:** 10.1186/s40635-019-0251-4

**Published:** 2019-05-22

**Authors:** Nicholas L. Jackson Chornenki, Robert Coke, Andrew C. Kwong, Dhruva J. Dwivedi, Michael K. Xu, Ellen McDonald, John C. Marshall, Alison E. Fox-Robichaud, Emmanuel Charbonney, Patricia C. Liaw

**Affiliations:** 1 Department of Medicine, McMaster University, Hamilton, ON Canada; 2 Thrombosis and Atherosclerosis Research Institute, Hamilton, ON L8L 2X2 Canada; 3 Departments of Surgery and Critical Care Medicine, St. Michael’s Hospital, University of Toronto, Toronto, ON Canada; 4 Department of Critical Care Medicine, Hôpital du Sacré-Coeur de Montreal and Hôpital de Trois-Rivières, University of Montreal, Montreal, QC Canada

**Keywords:** Cell-free DNA, protein C, traumatic injury, NETosis

## Abstract

**Background:**

Circulating cell-free DNA (cfDNA) may contribute to the pathophysiology of post-injury inflammation and coagulation in trauma. However, the source and mechanism of release of cfDNA in trauma is not well understood. One potential source of cfDNA is from Neutrophil Extracellular Traps (NETs), released by activated neutrophils during the process of NETosis. The primary objective of our study was to determine if cfDNA has prognostic utility in trauma. The secondary objective of this study was to determine the source of cfDNA in trauma compared to sepsis.

**Methods:**

We studied trauma patients from two prospective observational cohort studies: the DNA as a Prognostic Marker in ICU Patients (DYNAMICS) study and the Endotoxin in Polytrauma (ENPOLY) study. We also studied septic patients from the DYNAMICS study. Citrated plasma samples were collected longitudinally from the patients (days 1 to 7). The following molecules were measured in the plasma samples: cfDNA, protein C (PC), myeloperoxidase (MPO) (a marker of neutrophil activation), citrullinated Histone H3 (H3Cit, a marker of NETosis), cyclophilin A (a marker of necrosis), and caspase-cleaved K18 (a marker of apoptosis).

**Results:**

A total of 77 trauma patients were included (*n* = 38 from DYNAMICS and *n* = 39 from ENPOLY). The median age was 49 years; 27.3% were female, and mortality was 16.9% at 28 days. Levels of cfDNA were elevated compared to healthy values but not significantly different between survivors and non-survivors. There was a positive correlation between MPO and cfDNA in septic patients (*r* = 0.424, *p* < 0.001). In contrast, there was no correlation between MPO and cfDNA in trauma patients (*r* = – 0.192, *p* = 0.115). Levels of H3Cit, a marker of NETosis, were significantly elevated in septic patients compared to trauma patients (*p* < 0.01) while apoptosis and necrosis markers did not differ between the two groups.

**Conclusion:**

Our studies suggest that the source and mechanism of release of cfDNA differ between trauma and sepsis patients. In sepsis, cfDNA is likely primarily released by activated neutrophils via the process of NETosis. In contrast, cfDNA in trauma appears to originate mainly from injured or necrotic cells. Although cfDNA is elevated in trauma and sepsis patients compared to healthy controls, cfDNA does not appear to have prognostic utility in trauma patients.

**Trial registration:**

ClinicalTrials.gov Identifier: NCT01355042. Registered May 17, 2011

**Electronic supplementary material:**

The online version of this article (10.1186/s40635-019-0251-4) contains supplementary material, which is available to authorized users.

## Background

Traumatic injury causes approximately 5.1 million deaths per year and is a substantial contributor to morbidity worldwide [1–3]. Accurate identification of patients at greatest risk of death from trauma is dependent upon identifying effective predictors. Clinical scoring systems have been developed such as the Injury Severity Score [4] and the Revised Trauma Score [5] for critically ill patients. While these scores are useful for predicting mortality, they reflect external manifestations rather than underlying pathophysiologic mechanistic causes of organ dysfunction. Understanding the underlying pathophysiology in trauma is critical to the development and use of targeted interventions.

Over the past several years, circulating cell-free DNA (cfDNA) has been suggested to contribute to the pathophysiology of post-injury inflammation and coagulation in trauma [6, 7]. The release of cfDNA is presumed to occur via mechanical injury and tissue necrosis. Alternatively, cfDNA can also be released via NETosis, a unique form of cell death whereby activated neutrophils release neutrophil extracellular traps (NETs) in response to sterile inflammation, infection, or hypoxia [6, 8]. NETs are web-like networks of decondensed nuclear DNA in association with histones, granule proteins (e.g. myeloperoxidase), and antimicrobial peptides. A key signaling event in NETosis is the decondensation of chromatin by peptidylarginine deiminase 4 (PAD4), a nuclear enzyme that converts arginine residues to citrulline on histones. Unlike accidental cell death (necrosis), NETosis is a regulated cell death pathway and thus can potentially be modulated pharmacologically. For example, small molecular inhibitors of PAD4 can impair NET formation in mice [9].

cfDNA is the major structural element of NETs. The pathological effect of cfDNA is proposed to reflect its ability to trigger blood coagulation as well as to inhibit clot lysis which may lead to microvascular thrombosis [6, 10]. Furthermore, mitochondria-derived cfDNA promotes inflammation via activation of Toll-like receptor-9 (TLR-9) [11]. Although cfDNA has been shown to be elevated in trauma patients [11–13], the mechanisms by which it is released into the circulation are unknown. In addition, although cfDNA has been reported to be a predictive marker of outcome in trauma patients, the sample sizes in the studies are small and there is variation in the methods of DNA processing and quantification [14].

In this study, the primary objective was to examine the prognostic utility of cfDNA in trauma patients. The secondary objective was to investigate the source and mechanism of release of cfDNA in trauma compared to sepsis.

## Methods

### Study design and population

A total of 77 trauma patients were included from two primary studies. The Endotoxin in Polytrauma (ENPOLY) study [15], a single-center cohort study that recruited patients between April 2010 and April 2012 at St Michael’s Hospital in Toronto, ON, and the DNA as a Prognostic Marker in ICU Patients (DYNAMICS) study, a multicentre pan-Canadian observational study that recruited patients between November 2010 and January 2013 [16]. The inclusion criteria for the ENPOLY trauma study were Injury Severity Score (ISS) of 16 or greater, enrolled within 24 h of admission, and without evidence of infection. DYNAMICS trauma patients included in this cohort were classified as multiple trauma with an episode of shock who were expected to remain in the ICU for ≥ 72 h (shock must have been present within the previous 24 h and may have resolved at the time of enrolment). Shock was defined as SBP ≤ 90 or MAP ≤ 65 mmHg or a decrease in SBP of 40 from baseline, or lactate > 1.5 times the upper limit of normal.

Septic patients from the DYNAMICS had a confirmed or suspected infection on the basis of clinical data at the time of screening, at least one dysfunctional organ system, 3 or more signs of systemic inflammatory response syndrome (SIRS), and were expected to remain in the ICU for ≥ 72 h. The presence of organ dysfunction was defined by (1) SBP ≤ 90 mmHg or MAP ≤ 65 mmHg for at least 1 h despite fluid resuscitation, adequate intravascular volume status, or use of vasopressor in attempt to maintain systolic BP ≥ 90 or MAP ≥ 65 mmHg, (2) P/F ratio ≤ 250 in the presence of other dysfunctional organs or systems, or ≤ 200 if lung is the only dysfunctional organ, (3) acute rise in creatinine > 171 mM or urine output < 0.5 ml/kg body weight for 1 h despite adequate fluid resuscitation, (4) unexplained metabolic acidosis (pH ≤ 7.30 or base deficit ≥ 5 with lactate > 1.5 times the upper limit of normal, and (5) platelet count < 50,000 or a 50% drop over the 3 days prior to ICU admission. Plasma was collected from healthy volunteers as a healthy control and stored in the same manner as the sepsis and trauma samples. Of the 26 healthy controls, 42.3% were female and the median age was 32 (range 19–60). No attempt was made to match healthy controls for age or sex with sepsis or trauma samples.

### Clinical data

Data was collected with respect to demographics, dates of admission and discharge, and 28-day in-hospital mortality, and routine clinical variables. The Multiple Organ Dysfunction Score (MODS) [17] was used to quantify organ dysfunction.

### Measurements of cfDNA and protein C levels in plasma samples

Measurements of cfDNA and protein C were performed using frozen patient plasma samples collected daily during the patients stay in hospital. In order to measure cfDNA from plasma, cfDNA was first isolated from 250 μl of plasma with a QIAamp DNA Blood Mini Kit (Qiagen, Valencia, CA, USA). The concentration of DNA was measured using a spectrophotometer (Beckman DU 7400; Beckman Coulter Inc., Brea, CA, USA). Protein C levels were measured using an ELISA (Affinity Biologicals Inc., Ancaster, ON, Canada).

### MPO and cell death assays

Citrullinated Histone H3 (H3Cit) was quantified as a NETosis marker using methodology previously described [18]. Neutrophil myeloperoxidase (MPO) assay was performed using a human Myeloperoxidase DuoSet ELISA (R&D Systems, Minneapolis, Mn, USA). The levels of a caspase-cleaved cytokeratin-18 (CK18), a marker of apoptotic cell death [19], were measured using an ELISA (Peviva AB, Bromma, Sweden). Necrosis was quantified by measuring levels of cyclophilin A using an ELISA, a cytosolic protein isomerase that is a released by necrotic cell death when the integrity of the plasma membrane is compromised (RayBiotech, GA) [20].

### Statistical analysis

Continuous values were reported as median (range), and proportions were reported as percentages. Differences in demographic and baseline variables were determined with Mann-Whitney or Fisher’s exact test as appropriate. Statistical analysis of PC and cfDNA over time between survivors and non-survivors was performed using students Mann-Whitney *U* test with a correction for multiple comparisons. Spearman’s correlation coefficient was utilized for correlation analysis due to the non-Gaussian distribution of data. Analysis was performed using GraphPad Prism 7 (GraphPad Software, La Jolla, CA, USA).

## Results

### Patient characteristics

We included 77 trauma patients in our study (38 from the DYNAMICS study, 39 from the ENPOLY study). The patients were recruited from six tertiary Canadian ICUs (DYNAMICS) and from a single tertiary center (ENPOLY). The 28-day mortality rate in our cohort was 16.9%. Baseline characteristics of the patients are shown in Table 1. Non-survivors were significantly more likely to be on vasopressors during day 1 and had lower platelet count and higher MODS scores.

**Table 1:** Baseline characteristics of 77 trauma patients

	Overall (77)	Survivors (64)	Non-survivors (13)	*p* value
Age (years)	49.2 (16–100)	47 (16–100)	66 (31–90)	0.0063
Female gender	27.3%	25%	38.5%	0.33
Vasopressor use on day 1	28.6%	23.4%	53.8%	.042
Baseline creatinine (μg/dL)	87 (45–597)	86 (45–597)	101 (49–155)	0.60
White blood cell count at admission (1.0 × 10^9^/L)	11 (3–37.9)	10.9 (3–31.7)	14 (6–37.9)	0.23
Day 1 lactate level (mM)	3.15 (0.9–15.4)	3.0 (0.9–13.6)	4.0 (1.3–15.4)	0.15
Day 1 platelet count (1.0 × 10^9^/L)	141.5 (21–472)	166 (68–472)	114(21–228)	< 0.001
Head injury	46.75%	46.88%	46.15%	.99
Total fluids during first 24 h (mL)	5093 (484–37550)	5093 (484–37550)	5148 (679–19000)	0.88
Temperature on admission (C°)	37.4 (31.8–40.5)	37.5 (31.8–40.5)	35.7 (33.0–39.2)	0.22
Baseline MODS	6 (0–14)	5 (0–14)	7 (5–11)	.007
Maximal MODS	8 (0–14)	7 (0–14)	9 (5–14)	0.015

Note: Results are displayed as median with range. *MODS* = Multiple Organ Dysfunction Score

### cfDNA and PC in trauma patients

**Fig. 1:**
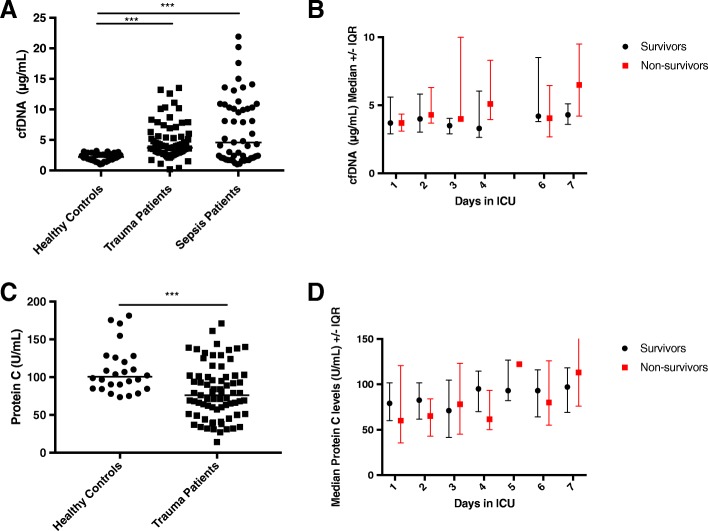
cfDNA and PC levels in trauma patients. **a** Median cfDNA levels in trauma patients, septic patients, and healthy controls. **b** Median and IQR temporal changes in levels of cfDNA in trauma survivors and non-survivors. **c** Median PC levels in trauma patients and healthy controls. **d** Median and IQR temporal changes in levels of PC in trauma survivors and non-survivors. Note: ***p < 0.001. IQR = interquartile range

The median baseline level of cfDNA in trauma patients, while lower than septic patients was significantly higher compared with healthy volunteers (Fig. 1a). However, levels of cfDNA did not differ significantly between survivors and non-survivors at any time points (Fig. 1b). Since mortality in trauma is thought to be associated in part with a consumptive coagulopathy, we also measured levels of protein C (PC), a naturally occurring anticoagulant. Plasma levels of PC in trauma patients were significantly lower than healthy volunteers on day 1 (Fig. 1c), although levels between survivors and non-survivors did not significantly differ at any time point (Fig. 1d). Comparison of our spectrophotometry method of cfDNA quantification to qPCR quantification methodology is shown in Additional file 2. Spectrophotometry was the more sensitive technique.

### Correlation between cfDNA and organ dysfunction

Previous studies have shown that plasma levels of cfDNA are elevated in septic mice and that administration of recombinant DNase1 (which digests DNA) reduces organ damage and improves outcomes [21]. To evaluate the possibility that high cfDNA levels would lead to a greater degree of organ dysfunction in trauma patients, we computed delta-MODS (the difference between MODS on day 1 and maximal MODS for each trauma patient. There was no significant relationship between initial cfDNA and delta-MODS (*r* = − 0.1478, *p* = 0.203), maximal MODS, or day 1 MODS, suggesting that in this trauma population, the day 1 value of cfDNA does not correlate with organ dysfunction.

### Investigation of the source and mechanism of release of cfDNA in trauma and septic patients

**Fig. 2:**
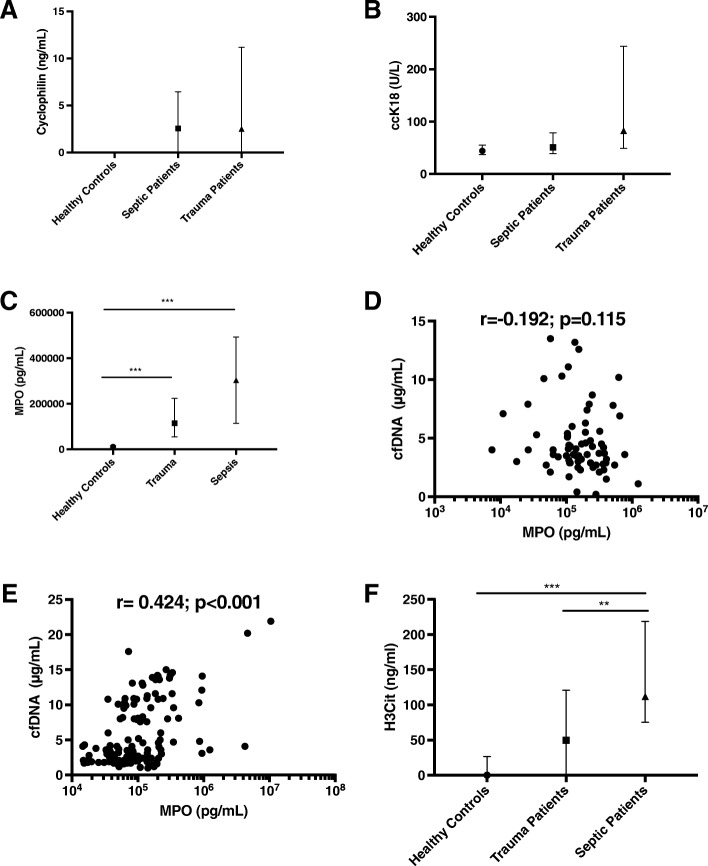
Investigations into the potential sources of cfDNA. **a** Necrosis as quantified by levels of cyclophilin A. **b** Apoptosis as quantified by ccK18 fragments. **c** Day 1 MPO levels in sepsis, trauma, and healthy controls. **d** Correlation between myeloperoxidase (MPO) and cell-free DNA (cfDNA) in trauma patients. **e** Correlation between MPO and cfDNA levels in septic patients. **f** Levels of citrullinated histone H3 in sepsis, trauma, healthy controls. Note: ***p < 0.001, **p < 0.01; data represent median and interquartile range

We next investigated the source and mechanism of release of cfDNA in trauma patients in comparison to septic patients. The baseline characteristics of 49 septic patients from the DYNAMICS study are shown in Additional file 1. As shown in Fig. 2a, b, markers of necrosis (cyclophilin A) [20] and apoptosis (ccK18) [19] did not significantly differ between septic and trauma samples. We next quantified levels of myeloperoxidase (MPO) which is a neutrophil enzyme that is released during NETosis. Day 1 MPO levels were significantly higher in trauma and sepsis patients than in healthy controls. In our population of trauma patients, the day 1 MPO level was not correlated with day 1 cfDNA levels (*r* = – 0.192, *p* = 0.115) (Fig. 2d). However, in septic patients from the DYNAMICS study, MPO level measured on day 1 was strongly correlated with day 1 cfDNA (*r* = 0.424, *p* < 0.001) (Fig. 2e), suggesting that cfDNA in septic patients is released by neutrophils via the process of NETosis. MPO levels were not significantly associated with neutrophil count suggesting that these differences are not due to absolute differences in cell numbers.

To further investigate the potential contribution of NETosis to cfDNA levels, we quantified levels of citrullinated histone H3 (H3-Cit) which is a PAD4-mediated post-translational modification that is a hallmark of NETosis [18]. Plasma levels of H3-Cit were measured in trauma patients, septic patients, and healthy controls. Day 1 levels of H3-Cit were significantly higher in septic patients compared to trauma patients or healthy controls (Fig. 2f). These results suggest that cfDNA is released via NETosis in septic patients but not in trauma patients.

## Discussion

This retrospective observational combined cohort study examined the prognostic utility and source of cfDNA in trauma and sepsis patients. cfDNA did not have prognostic utility in our trauma population. Levels of H3Cit, a marker of NETosis, were significantly higher in septic patients than in trauma patients. Furthermore, there was a positive correlation between cfDNA and neutrophil-derived MPO, an enzyme released during NETosis in sepsis patients but not in trauma patients. Taken together, these results show a difference in the involvement of NETosis to cfDNA source between sepsis and trauma which may contribute to the discrepancy in prognostic utility of cfDNA.

Although the average day 1 cfDNA levels in the trauma population were elevated compared to healthy controls, our results do not support previous findings that cfDNA has prognostic utility in trauma [14, 22]. Variations in the quantification methods between our study and previous studies likely account for this. Our method of cfDNA measurement is based on spectrophotometric absorbance at 260 nm, and thus measures the total amount of cfDNA, irrespective of the source or fragmentation state. This is the same methodology we used to quantify cfDNA in septic patients [23]. Previous studies that show prognostic value for cfDNA in trauma used either a PCR-based approach that detects a single gene [12, 24–27] or a fluorescence approach [28, 29]. The PCR-based method detects approximately 10-fold less DNA compared to our method, and the amount quantified is impacted by DNA fragmentation [11, 23]. Our method of quantification provides a more robust and accurate measure of plasma cfDNA levels.

With respect to the difference in prognostic utility of cfDNA between trauma patients and what we previously reported in sepsis [23], this is likely due to variation in the source of cfDNA. Our data suggests that cfDNA in septic patients originates from neutrophils via NETosis. Our study is complemented by a recent analysis of methylation profiles of cfDNA which suggest that in sepsis the DNA is predominantly of neutrophil origin compared to healthy individuals [30]. In contrast, while NETosis may occur in trauma, cfDNA in trauma patients appears to be mainly released from injured or necrotic cells. In addition to releasing cfDNA, NETosis also releases neutrophil elastase (NE). NE has been shown to degrade antithrombin and TFPI, thereby impairing natural anticoagulant mechanisms [31–33] NE is also capable of causing tissue damage which reduces the ability of a host to fight infection [34]. The damaging effects of NE released by NETosis may explain why cfDNA appears to be more harmful and thus have prognostic utility in sepsis but not in trauma.

Both necrosis and apoptosis also occurred in sepsis and trauma samples, suggesting that multiple mechanisms of cell death contribute to the total amount of circulating cfDNA. Importantly, processes of cell death such as necrosis are caused by mechanical and chemical insults and cannot be reversed by molecular intervention. In contrast, regulated pathways such as NETosis can potentially be modulated pharmacologically. In a mouse model, PAD4 inhibition was sufficient to disrupt NET formation [9]. Thus, as a regulated and therefore potentially therapeutically targetable process, NETosis may represent a potential therapeutic target in sepsis.

We previously reported that plasma levels of cfDNA in septic non-survivors were more than 3-fold higher than that in septic survivors [23]. Previous reports in small sample size of often only two or three trauma non-survivors have shown 2-fold or higher levels of cfDNA in non-survivors [14]. In this investigation, cfDNA does not have prognostic utility in trauma. Our sample size of 13 non-survivors and 64 survivors allows > 80% power to detect an increase in cfDNA among non-survivors of 50%. Thus, our sample size was sufficient. A limitation of our study is that we cannot exclude the possibility that additional forms of cell death such as necroptosis contributed to producing cfDNA [35]. Another limitation of our study is that we are not able to distinguish between nuclear and mitochondrial DNA based on Qiagen-based DNA isolation methods.

## Conclusions

Our studies suggest that the source and mechanism of release of cfDNA differ between trauma and sepsis patients. In sepsis, cfDNA is likely primarily released by activated neutrophils via the process of NETosis. In contrast, cfDNA in trauma appears to originate mainly from injured or necrotic cells. The lack of contribution from NETosis to cfDNA may account for why in our study total cfDNA did not have prognostic value in trauma patients.

## Additional files

Additional file 1: **Table S1.** Baseline characteristics of 49 septic patients. (DOCX 16 kb)

Additional file 2: **Table S2.** Comparison of methods of cfDNA quantification. (DOCX 17 kb)

## Abbreviations

cfDNA: Cell-free DNA
H3Cit: Citrullinated Histone H3
MPO: Myeloperoxidase
NETs: Neutrophil extracellular traps
