# Supplementary material for: Comparison of the source and prognostic utility of cfDNA in trauma and sepsis
Source: Intensive Care Med Exp. 2019 May 22;7:29. doi: 10.1186/s40635-019-0251-4 (PMC6531595; doi:10.1186/s40635-019-0251-4)
Supplement: Supplementary file 1 — Table S1. Baseline characteristics of 49 septic patients. (DOCX 16 kb) [file 40635_2019_251_MOESM1_ESM.docx]

**Additional file 1: Table S1.** Baseline characteristics of 49 septic patients

|  |  |
| --- | --- |
| Age (years) | 66.2 (28.4–88.6) |
| Female Gender | 34.7% |
| Mortality rate | 35.0% |
| Vasopressor use on day 1 | 63.3% |
| Baseline Creatinine (µg/dL) | 96 (28–513) |
| White Blood Cell Count at admission | 13.0 (0.1–51) |
| Baseline lactate level (mM) | 3.0 (0–15) |
| Day 1 platelet count | 165 (10–507) |
| Day 1 PC (U/ml) | 59 (5–199) |
| Day 1 cfDNA (ug/ml) | 5.0 (1.0–22.0) |
| Total fluids during first 24h (mL) | 9795 (1307­–27687) |
| Baseline MODS | 7 (0–17) |

***Note****: data are presented as percentage or median and range.*
