# Supplementary material for: Comparison of the source and prognostic utility of cfDNA in trauma and sepsis
Source: Intensive Care Med Exp. 2019 May 22;7:29. doi: 10.1186/s40635-019-0251-4 (PMC6531595; doi:10.1186/s40635-019-0251-4)
Supplement: Supplementary file 2 — Table S2. Comparison of methods of cfDNA quantification. (DOCX 17 kb) [file 40635_2019_251_MOESM2_ESM.docx]

***Comparison of methods of cfDNA quantification.***

We analysed three septic non-survivor samples using both the method described in the main manuscript and with qPCR. QPCR was performed using cell-free DNA isolated from Septic plasma from survivors and non-survivors. Primers were those Beta-globinY354F: 5’-GTG CAC CTG ACT CCT GAG GAG A-3’, Beta-globinY455R: 5’-CCT TGA TAC CAA CCT GCC CAG-3’. The samples were probed for beta-globin using SYBR-green. From the QPCR results we calculated the Genome equivalents (GE). The GE values were then converted to ug/mL. The results in Supplemental Table 2 demonstrate that the qPCR is less sensitive method for measuring cell-free DNA levels. The amount of DNA quantified using the spectrophotometer is substantially higher than that quantified by PCR.

**Additional file 2: Table S2.** Comparison of methods of cfDNA quantification

|  | *Spectrophotometer (ug/mL)* | *QPCR (ug/mL)* |
| --- | --- | --- |
| *Non-survivor 1* | 5.65 | 0.05 |
| *Non-survivor 2* | 11.40 | 0.37 |
| *Non-survivor 3* | 58.50 | 0.22 |
